# Factors associated with four atypical cases of congenital syphilis in England, 2016 to 2017: an ecological analysis

**DOI:** 10.2807/1560-7917.ES.2017.22.49.17-00750

**Published:** 2017-12-07

**Authors:** Martina Furegato, Helen Fifer, Hamish Mohammed, Ian Simms, Paul Vanta, Sharon Webb, Kirsty Foster, Margaret Kingston, André Charlett, Bhavita Vishram, Claire Reynolds, Noel Gill, Gwenda Hughes

**Affiliations:** 1 HIV & STI Department, Public Health England, Colindale, London, United Kingdom; 2 Bacteriology Reference Department, National Infection Service, Public Health England, Colindale, London, United Kingdom; 3 NHS Infectious Diseases in Pregnancy Screening Programme, PHE Screening, London, United Kingdom; 4 The Northern Integrated Contraception, Sexual Health & HIV Service, Manchester University NHS Foundation Trust, Manchester, United Kingdom; 5 Statistics, Modelling and Economics Department, National Infection Service, Public Health England, Colindale, London, United Kingdom; 6 NHS Blood & Transplant/PHE Epidemiology Unit, Public Health England, Colindale, London, United Kingdom

**Keywords:** United Kingdom, sexually transmitted infections, syphilis, antenatal screening, pregnancy, public health policy, surveillance, women's health, epidemiology, statistics

## Abstract

Four isolated cases of congenital syphilis born to mothers who screened syphilis negative in the first trimester were identified between March 2016 and January 2017 compared with three cases between 2010 and 2015. The mothers were United Kingdom-born and had no syphilis risk factors. Cases occurred in areas with recent increases in sexually-transmitted syphilis among women and men who have sex with men, some behaviourally bisexual, which may have facilitated bridging between sexual networks.

Since 2011, the rapid increase in sexually transmitted infectious syphilis (ST-syphilis) (primary, secondary and early latent) diagnoses among gay, bisexual or other men-who-have-sex-with-men (MSM) in England has contrasted with a low, relatively stable number among heterosexuals [1,2]. Congenital syphilis (CS) is rare in England with only 21 cases occurring between February 2011 and January 2017 (data not shown) and [3]. Cases have been associated with mothers who were socially marginalised and encountered barriers when accessing antenatal care. Between March 2016 and January 2017 inclusive, we identified four apparently atypical CS cases that were caused by incident maternal syphilis infection after a negative first trimester antenatal screen. We set out to investigate whether this was an unusual event and if there was a spatiotemporal relationship between the epidemiology of ST-syphilis and the recent CS cases to inform the development of a targeted public health response.

## Data sources and analyses

Data were obtained from reports of CS made to Public Health England (PHE) National Infection Service; diagnoses of infectious syphilis reported to the genitourinary medicine clinic activity dataset (GUMCAD) sexually transmitted infections (STI) surveillance system, and mid-year population estimates for England [4,5]. Three analyses were undertaken: simulation modelling with 100,000 simulations based on a Poisson distribution of CS cases reported since 2010; time-series analyses (TSA) were used to identify exceedances in ST-syphilis case-frequencies by sex, sexual orientation and area for the period from January 2011 to September 2016 [6]. The 152 English upper-tier local authorities (LA) were categorised into one of following syphilis epidemiological areas (SEA): (i) incident areas: LAs where the mothers of the CS cases lived; (ii) endemic areas: LAs with established spatiotemporal clusters of ST-syphilis in men [6]; (iii) rest of England: all other LAs in England.

Pearson’s chi-squared and Kruskal-Wallis tests were used to compare behavioural and demographic characteristics of ST-syphilis cases across SEAs. To avoid circular analysis bias the mothers of the CS cases were removed from the denominator.

## Case characteristics

The four CS cases were considered unusual because their mothers had screened negative in early pregnancy. The screening used treponemal total antibody (IgG/IgM) tests. In the subsequent investigation of all four cases, the stored early screening samples, together with maternal samples taken at the time of the CS diagnoses, were tested at the PHE national Reference laboratory using a treponemal total antibody enzyme immunoassay (EIA), IgM specific EIA, *Treponema pallidum* particle agglutination and Rapid Plasma Reagin tests. It was confirmed that maternal syphilis had been acquired at some point during pregnancy, after negative early screening. No syphilis risk factors were identified by their physicians at the time of screening. For two cases, our investigations identified social vulnerabilities that had been highlighted in previous studies [3]. All four mothers were white and born in the United Kingdom (UK).

Two infants were classified as confirmed cases of CS (PCR-positive) following the European Union case definition [7]. The remaining two were classified as probable cases because it was likely that the infants’ antibody responses had been attenuated due to late acquisition of maternal syphilis and early antibiotic treatment.

**Figure 1:**
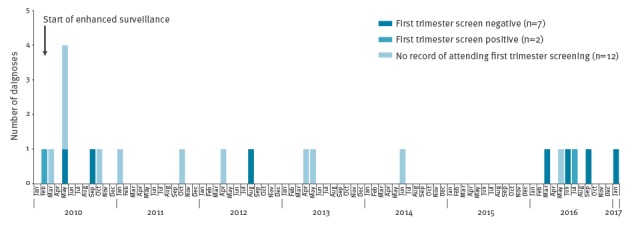
Congenital syphilis cases by month and year of birth and result of first trimester screening, England, February 2010–January 2017 (n=21)

Since 2010 there have been 21 CS cases reported in England. Nine of these mothers had a record of antenatal screening of whom seven (including the 4 recent cases) had screened negative at their first trimester antenatal screen (Figure 1) [3]. The probability of observing four CS cases born to screen-negative mothers (‘screen-negative’ cases) in a 10-month period was estimated to be less than 1%.

Cases were defined as confirmed following [3].

## Spatiotemporal variation

From January 2011 to December 2016, ST-syphilis diagnosis rates (per 100,000 population) in incident areas in England, increased by 130% from 1.1 to 2.3, in heterosexual women and by 52% from 8.9 to 15.5, in MSM, but fell by 37% from 4.3 to 2.1, in heterosexual men. In endemic areas, rates fell by 42% from 3.6 to 2.1, in heterosexual women, but rose by 131% from 37.6 to 86.8) in MSM and by 21% (5.2 to 6.3) in heterosexual men. In the rest of the country, rates rose by 19% from 1.6 to 1.9 in heterosexual women, by 138% from 5.0 to 11.9 in MSM and by 18% from 2.7 to 3.2 in heterosexual men.

**Figure 2:**
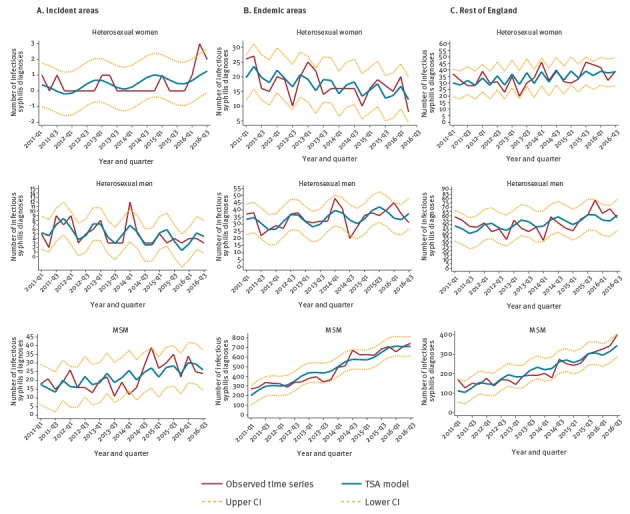
Time trend and time series analysis model for sexually transmitted infectious syphilis diagnoses by sexual orientation and syphilis epidemiological areas, England, January 2011–September 2016

CI: 95% confidence interval; TSA: time series analysis.

Between March 2016 and January 2017, the number of ST-syphilis cases in heterosexual women significantly exceeded the upper confidence interval in the incident areas (Figure 2). No other exceedance in ST-syphilis cases was identified either for the other population groups or other geographic areas analysed.

In 2016, the proportion of heterosexual women diagnosed with ST-syphilis who were UK-born was greater in the incident areas (2/3) and in the rest of the country (98/120) compared with the endemic areas (13/37; p < 0.001). The proportion of MSM diagnosed with ST-syphilis who were behaviourally bisexual was greater in the incident areas (4/29) compared with endemic areas (11/2,113) and the rest of England (106/1,122; p < 0.001).

## Discussion

Our ecological analyses cannot be used to infer the mothers’ risk factors in the specific cases analysed here. Nevertheless, while numbers were small, the rapid increase in syphilis cases in women and MSM and the relatively high proportion of behaviourally bisexual MSM suggest there may have been increased opportunities for transmission between sexual networks in the incident areas.

Syphilis remains rare in the general population: population-based blood donation data show that between 2012 and 2016, the rate of recent syphilis infection in the UK was 1.1 per 100,000 donations from men and 0.5 per 100,000 donations from women [8]. Low rates of CS are also seen in many other European countries which suggest that most have programmes that aim to eliminate CS [9]. The incidence of CS in the UK remains below the World Health Organization (WHO) elimination threshold (≤ 0.5/1,000) and the measures of health service provision recommended by WHO have been achieved [3,10]. Nevertheless, the recent cases highlight the continuing clinical and social significance of this rare but important disease. CS is preventable through screening and treatment of pregnant women with a single injection of benzathine benzylpenicillin [10,11]. However, creating a strategy to eliminate mother-to-child transmission is challenging in the face of the rising syphilis incidence within the population. Uptake of antenatal syphilis screening in England is high at 97% [3]. Guidelines recommend that women identified as being at risk be re-screened in the third trimester [12]. However, as shown here, it can be difficult to identify those at risk and routine third trimester screening is unlikely to be cost-effective [13].

Maintaining the integrity of clinical care pathways to ensure timely treatment, management and partner notification of identified cases is therefore crucial, and should be subject to regular review [14-16]. Reviewing the performance of these pathways together with clinical outcomes will form part of a new Infectious Disease in Pregnancy Screening Integrated Outcomes System of Maternal and Paediatric Surveillance that is being extended to include syphilis in 2018 [17].

These CS cases emphasise the need for women to be aware of maintaining their sexual health throughout pregnancy. Midwives and other key healthcare professionals play an important role in raising awareness among mothers and their partners to sexual health throughout pregnancy. To support this, the English National Infectious Diseases in Pregnancy Screening Programme is developing media and resources including a bespoke professional e-learning package; ‘in-consultation’ counselling resource with guidance for practitioners, updated midwifery resource cards and information leaflets for screen-positive women [18,19].

Since the re-emergence of ST-syphilis at the beginning of the century the epidemic has been focused on MSM. Controlling syphilis in MSM and the spread to other population groups is a public health priority. Efforts should be multi-factorial, including risk reduction behavioural interventions as well as improving syphilis testing coverage and frequency in those at greatest risk, including HIV-positive MSM.
